# Vasculitis and familial Mediterranean fever: Description of 22 French adults from the juvenile inflammatory rheumatism cohort

**DOI:** 10.3389/fmed.2022.1000167

**Published:** 2022-10-28

**Authors:** Salam Abbara, Jean-Benoit Monfort, Léa Savey, Philippe Moguelet, David Saadoun, Claude Bachmeyer, Olivier Fain, Benjamin Terrier, Zahir Amoura, Alexis Mathian, Laurent Gilardin, David Buob, Chantal Job-Deslandre, Jean-François Dufour, Rebecca Sberro-Soussan, Gilles Grateau, Sophie Georgin-Lavialle

**Affiliations:** ^1^Département de Médecine Interne, Centre de Référence des Maladies Auto-Inflammatoires et des Amyloses d’Origine Inflammatoire (CEREMAIA), Hôpital Tenon, Sorbonne Université, AP-HP, Paris, France; ^2^Département de Dermatologie, Hôpital Tenon, Sorbonne Université, AP-HP, Paris, France; ^3^Département d’Anatomo-Pathologie, Hôpital Tenon, Sorbonne Université, AP-HP, Paris, France; ^4^Département de Médecine Interne et Immunologie Clinique, Centre National de Référence Maladies Autoimmunes Systémiques Rares, Centre National de Référence Maladies Autoinflammatoires et Amylose Inflammatoire, INSERM UMR_S 959, Immunologie-Immunopathologie-Immunotherapie, i3 and Département Hospitalo-Universitaire Inflammation-Immunopathologie-Biothérapie i2B, Groupe Hospitalier Pitié-Salpêtrière, Sorbonne Université, AP-HP, Paris, France; ^5^Service de Médecine Interne, Hôpital Saint-Antoine, Sorbonne Université, APHP, Paris, France; ^6^Service de Médecine Interne, Centre de Référence Maladies Systémiques et Autoimmunes Rares d’Ile de France, Hôpital Cochin, Université Paris Cité, AP-HP, Paris, France; ^7^Service de Médecine Interne 2, Institut E3M, Inserm UMRS, Centre d’Immunologie et des Maladies Infectieuses (CIMI-Paris), French National Referral Center for Systemic Lupus Erythematosus, Antiphospholipid Antibody Syndrome and Other Autoimmune Disorders, Groupement Hospitalier Pitié–Salpêtrière, Sorbonne Université, AP-HP, Paris, France; ^8^Département de Médecine Interne et Immunologie Clinique, Groupe Hospitalier Pitié-Salpêtrière, Sorbonne Université, AP-HP, Paris, France; ^9^Service de Pédiatrie, Immunologie, Hématologie et Rhumatologie, Centre de Référence pour les Rhumatismes Inflammatoires et les Maladies Auto-Immunes Systémique Rare de l’Enfant (RAISE), Hôpital Necker-Enfants Malades, AP-HP, Université Paris Cité, Paris, France; ^10^Service Médecine Interne, Hôpital Nord-Ouest, Centre Hospitalier Villefranche sur Saône, Gleize, France; ^11^Service de Transplantation Rénale Adulte, Hôpital Necker-Enfants Malades, AP-HP, Université Paris Cité, Paris, France; ^12^INSERM U938, Centre de Recherche Saint-Antoine (CRSA), Paris, France

**Keywords:** vasculitis, familial Mediterranean fever, polyarteritis nodosa, IgA vasculitis, pyrin, anti-neutrophil cytoplasmic antibody-associated vasculitis, Behçet syndrome

## Abstract

**Objective:**

The frequency of vasculitis may be increased in patients with Familial Mediterranean Fever (FMF), according to several studies. Our aim was to assess the characteristics of French adult patients with both diseases.

**Methods:**

Patients with vasculitis were selected from patients followed for FMF in the French JIR-cohort.

**Results:**

Twenty-two patients were included [polyarteritis nodosa (PAN) *n* = 10, IgA vasculitis *n* = 8, unclassified vasculitis *n* = 2, granulomatosis with polyangiitis *n* = 1, and microscopic polyangiitis *n* = 1]. Pathogenic mutations in exon 10 were found in all 21 patients (96%) for which *MEFV* testing results were available, and 18 (82%) had two pathogenic mutations. Histology showed vasculitis in 59% of patients. Most patients with FMF-associated PAN were HBV-negative and had an inactive FMF before PAN onset, and 40% had a peri-renal or central nervous system bleeding. Most patients with FMF-associated IgA vasculitis had an active FMF before vasculitis onset, and 25% had digestive bleeding. Both patients with unclassified vasculitis had ischemic and/or hemorrhagic complications.

**Conclusion:**

This study confirms the predominance of PAN and IgA vasculitis in patients with FMF and the high frequency of bleeding in FMF-associated PAN. FMF should be considered in case of persistent symptoms and/or inflammatory syndrome despite vasculitis treatment in Mediterranean patients.

## Introduction

Familial Mediterranean fever (FMF) is the most common monogenic auto inflammatory disease, mainly affecting people from Mediterranean countries and associated with mutations in the *MEFV* gene (1). *MEFV* encodes pyrin, a protein expressed in neutrophils and monocytes and playing an important role in the innate immune response, resulting in the production of interleukin (IL)-1beta (2). Numerous reports suggest a higher frequency of vasculitis in patients with FMF compared to the general population (3–6). These frequencies could reach 2.7–7% for IgA vasculitis (3–5), 0.9–1.4% for polyarteritis nodosa (PAN) (4, 5), and 0.4% for Behçet disease (6). Moreover, the clinical characteristics of these vasculitis may differ between patients with FMF and the general population. Particularly, patients with FMF and PAN seem to have a higher incidence of peri-renal hematoma (7). Most studies arised from Turkey or the Middle East and data regarding European patients with vasculitis and FMF are lacking. In this nationwide French retrospective study, we report the main characteristics and outcomes of adult patients with FMF and vasculitis from the JIR-cohort.

## Methods

Patients with vasculitis diagnosed according to international criteria (8–11) were identified among patients aged >18 years in the French JIR-cohort with FMF (12). The Juvenile Inflammatory Rheumatism (JIR) cohort is an international multicenter prospective data repository for patients with systemic inflammatory or rheumatological disease^1^ (13). The following data were collected: Socio-demographic (age, sex, and ethnicity), background (family history, and comorbidities), FMF characteristics (diagnostic criteria, age at symptoms onset and at diagnosis, clinical manifestations, age at the start of colchicine treatment, C Reactive Protein (CRP) levels during flares and follow-up, FMF control before the onset of vasculitis, treatments, and dose of colchicine at vasculitis diagnosis), *MEFV* gene testing results (14, 15), vasculitis characteristics (age at diagnosis, clinical manifestations, histological results, results of arteriography and/or CT or MRI angiography, treatment, and follow-up). FMF control was judged on the presence of flares, and monitoring of CRP ± SAA, when available. Data are described as median (first quartile—third quartile) for continuous variables and number (%) for categorical variables. This observational study was based on data extracted from the JIR-cohort, established by the National Commission on Informatics and Liberty (CNIL, authorization number N0: 914677). Patients consented to be included in the JIR-cohort and were informed that data collected in medical records might be used for research studies in accordance with privacy rules.

## Results

Among 406 patients with FMF in the French JIR-cohort, 22 had vasculitis and were included (82% men). Most patients had a PAN (*n* = 10, 46%) or an IgA vasculitis (*n* = 8, 36%). The characteristics of FMF and of vasculitis in patients with PAN or IgA vasculitis are described in Table 1. Other patients had ANCA-associated vasculitis (*n* = 2 and 9%) or an unclassified vasculitis (*n* = 2 and 9%). The median ages at FMF and vasculitis diagnosis were 11.5 (7–22) and 22 (16.5–36.5) years, respectively. At least one episode of bleeding (renal, central nervous system, and pulmonary) or thrombosis complicated the vasculitis of 8 (36%) and 2 (9%) patients, respectively.

**TABLE 1:** Main characteristics of patients with FMF-associated PAN and IgA vasculitis.

	PAN (*n* = 10)	IgA vasculitis (*n* = 8)
Men	10 (100)	5 (63)
**FMF characteristics**		
Age at onset/diagnosis (years)	5 (–9)/12 (10–34)	7 (4–12)/9 (5–15)
*MEFV* pathogenic variants*		
M694V/M694V	7 (70)	6/7 (86)
M694V/V726A	1 (10)	–
I692del/I692del	1 (10)	–
M694V/−	1 (10)	–
M694I/−	–	1/7 (14)
Clinical manifestations**		
Fever	8/9 (89)	8 (100)
Abdominal pain	7/9 (78)	7 (88)
Thoracic pain	2/9 (22)	7 (88)
Arthralgia/arthritis	7/9 (78)	6 (75)
Myalgia	1/9 (11)	3 (38)
Pseudo erysipela	–	3 (38)
Testicular involvement	1/9 (11)	1 (13)
Onset before vasculitis	10 (100)	6 (75)
Colchicine/FMF control^$^ before vasculitis	10 (100)/7 (70)	4 (67)/1 (17)
**Vasculitis characteristics**		
Age at diagnosis (years)	36 (22.5–41.75)	13.5 (7.75–19.5)
**Clinical manifestations^$^**		
Cutaneous involvement	9 (90)	6/6 (100)
Renal involvement	3 (30)	5/6 (83)
Muscular involvement	7 (70)	2/6 (33)
Fever	6 (60)	1/6 (17)
Abdominal pain	6 (60)	3/6 (50)
Arthralgia/arthritis	5 (50)	3/6 (50)
Other^@^	2 (20)	1/6 (17)
Renal artery/cerebral aneurysms	3 (30)/1 (10)	–
Digestive bleeding	–	2/6 (33)
Histology compatible with the vasculitis^#^	8/8 (100)	3/3 (100)
**Treatment**		
Corticosteroids	6 (60)	3 (38)
Intravenous pulse cyclophosphamide	3 (30)	–
Other^†^	7 (70)	–

FMF, familial Mediterranean Fever; PAN, polyarteritis nodosa. Data are described as median (first quartile—third quartile) for continuous variables and number (%) for categorical variables.

*Data available for seven patients with IgA vasculitis.

**Data available for nine patients with PAN.

^$^Data available for six patients with IgA vasculitis.

^@^PAN: Weight loss, hypertension, and multineuritis (*n* = 1); weight loss, hypertension, oral aphthae, and testicular involvement (*n* = 1). IgA vasculitis: Weight loss (*n* = 1).

^#^Histology was available for eight patients with PAN and three patients with IgA vasculitis.

^†^Anakinra (*n* = 3), mycophenolate mofetil (*n* = 1), azathioprine (*n* = 1), plasma exchanges (*n* = 1), and non-steroidal anti-inflammatory drugs (*n* = 1).

### Polyarteritis nodosa and familial Mediterranean fever (*n* = 10)

Symptoms of FMF appeared during youth in most cases, with a median age at diagnosis of 11.5 (10–34) years. Three patients had a family history of FMF. FMF and AA amyloidosis were diagnosed concomitantly in one patient. *MEFV* testing results were available for all patients; 9/10 had two pathogenic mutations. All patients had ethnicities at risk of FMF (Jewish, *n* = 6; Turkish, *n* = 2; Arab, *n* = 1; and Armenian, *n* = 1). In all patients, FMF preceded vasculitis and low-dose colchicine was prescribed [median dose 1 (1–1) mg/day]. FMF was controlled in 8/10 patients at vasculitis onset.

**FIGURE 1:**
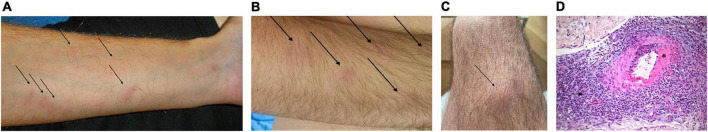
Picture of lesion and pathology slide in patients with PAN and FMF **(A–C)** subcutaneous nodules. **(D)** Skin biopsy (Hematoxylin, eosin, and saffron staining; magnification × 200): Deep dermal small artery vasculitis with intimal fibrinoid necrosis (*) and perivascular massive polymorphous inflammation of the adventice (arrow).

Most patients had an HBV-negative PAN (*n* = 9/10), with a median age at diagnosis of 36 (22.5–41.75) years. PAN was introduced by cutaneous signs (*n* = 6/10), peri-renal hematomas (*n* = 3/10), or multineuritis (*n* = 1/10). The main clinical manifestations of PAN were cutaneous (*n* = 9/10) or muscular involvement (*n* = 7/10), fever (*n* = 6/10), abdominal pain (*n* = 6/10), and arthralgia (*n* = 5/10). Cutaneous manifestations included subcutaneous nodules (*n* = 5/10, Figure 1), asymptomatic erythematous papules of the limbs (*n* = 3/10), purpura (*n* = 2/10), a pigmented livedo (*n* = 1/10), and an infiltrated, migrating, erythematous, pruritic annular rash (*n* = 1/10). No patient was tested for ADA2 deficiency. Histology was available for 8/10 patients and was compatible with PAN (Figure 1). Renal artery aneurysms were identified in 3/10 patients, and 4/10 patients had at least one episode of bleeding. Corticosteroids were administered to 6/10 patients. Notably, 3/10 patients with predominantly cutaneous involvement received anakinra, an IL-1-receptor antagonist, resulting in a rapid resolution of the clinical manifestations of PAN for all three patients, with resolution of the biological inflammatory syndrome for two patients. Treatment of PAN led to partial remission for all three patients with PAN introduced by a peri-renal hematoma. Despite treatment, these three patients had occasional flare-ups of febrile abdominal or joint pain, with an episode of purpura in one patient and an episode of erythema nodosum in another, but no patient had recurrent bleeding over a follow-up period of 3, 9, and 17 years. Follow-up was rarely longer than 1 year for the other patients. At last follow-up, 8/10 patients were taking colchicine at a median dose of 1 (1–2) mg/day, and 7/10 patients had a controlled FMF.

### IgA vasculitis and familial Mediterranean fever (*n* = 8)

The median age at FMF diagnosis was 9 (4.5–15) years. *MEFV* testing results were available for 7/8 patients: Six had two pathogenic mutations, and One had a single pathogenic mutation. One patient’s ethnicity was unknown; the others belonged to an ethnic group at risk of FMF (Jewish, *n* = 6 and Arab, *n* = 1). Five patients had a family history of FMF, while two and one patients had a family history of AA amyloidosis and Behçet disease, respectively. In 6/8 patients, the onset of FMF preceded that of vasculitis by a median interval of 4 (3–7) years. Only 4/6 patients were prescribed colchicine before vasculitis onset [median dose 2 (1.75–2) mg/day], and FMF was controlled in only 1/6 patients.

The median age at IgA vasculitis diagnosis was 13.5 (7.75–19.5) years. Most patients had a single episode (*n* = 5/8, 63%). Three patients had two (*n* = 1/8, 13%) or three (*n* = 2/8, 25%) episodes of vasculitis. Clinical manifestations of IgA vasculitis were available for 6/8 patients. The main ones were purpura (*n* = 6/6), renal involvement (*n* = 5/6, 83%), abdominal pain (*n* = 3/6, 50%), arthritis (*n* = 2/6, 33%), and muscular involvement (*n* = 2/6, 33%; pain *n* = 1 and myositis *n* = 1). For two patients, the vasculitis was complicated by digestive bleeding. Histology was rarely available (*n* = 3/8) and showed vasculitis (*n* = 3/3) and IgA deposits (*n* = 2/3). Three patients were treated with corticosteroids.

The median follow-up time after diagnosis of IgA vasculitis was 12.5 (0–26.5) years. At last follow-up, all patients were taking colchicine, at a median dose of 1.5 (1–2) mg/day, and half of the patients had an active FMF.

### Unclassified vasculitis and familial Mediterranean fever (*n* = 2)

One patient had a controlled FMF since his childhood, with two pathogenic mutations of *MEFV* (M694V/R761H). He developed a systemic vasculitis with a thrombosis of the superior mesenteric vein at age 36. Another patient presented with symptoms of FMF at age 13, with a homozygous M694V mutation. In the same year, he developed a vasculitis of vessels of all sizes. Despite treatments, he presented over the years several ischemic (stroke *n* = 1) or bleeding (bilateral peri-renal hematomas *n* = 1, testicular bleeding *n* = 1, and intra-alveolar hemorrhage *n* = 1) episodes, leading to his death at age 29. Both patients had two pathogenic mutations of *MEFV*.

### ANCA-associated vasculitis and familial Mediterranean fever (*n* = 2)

One patient had a FMF since the age of seven, with a heterozygous M694V mutation. He presented at age 17 with a constrictive pericarditis, arthromyalgia, an axonal sensory neuropathy on electromyography, erythema nodosum, a skin biopsy showing arteritis with thrombosis, and positive ANCA without specificity; the patient did not present renal involvement. He received corticotherapy and azathioprine. Few years later, he developed two unexplained episodes of stroke. The late positivity of MPO-ANCA antibodies at age 27 led to the diagnosis of ANCA-associated vasculitis. Persistence of fluctuating arthromyalgia, infiltrated papules with thrombosing and inflammatory vasculitis on biopsy, positive MPO-ANCA antibodies (maximum 33 IU/mL) and elevated CRP levels up to 35 mg/L led to the introduction of rituximab. Given the persistence of cutaneous-articular signs, elevated CRP levels, treatment with anakinra was initiated and resulted in resolution of the clinical manifestations.

Another patient had recurrent sinusitis with an alveolar hemorrhage and positive ANCA (type not specified), leading to the diagnosis of granulomatosis with polyangiitis. She was treated with oral corticosteroids and intravenous pulse cyclophosphamide. The persistence of a biological inflammatory syndrome despite treatment with intravenous pulse cyclophosphamide, then methotrexate, then mycophenolate mofetil, led to the diagnosis of FMF with a homozygous M694V mutation, which symptoms had occurred during childhood.

## Discussion

We describe the main clinical and genetical characteristics of 22 French adult patients with both FMF and vasculitis. The most frequent vasculitis were PAN (46%) and IgA vasculitis (36%), similar to previous reports from Mediterranean countries (4, 5, 7, 16). Despite possible overlaps between vasculitis and FMF clinical manifestations, all patients fulfilled the Tel Hashomer FMF criteria (12). A recent review pointed the paucity of genetic data for described patients with FMF and vasculitis (7). In this study, pathogenic mutations in exon 10 were found in all 21 patients for which *MEFV* testing results were available, and 18 had two pathogenic mutations (15).

Polyarteritis nodosa was the most frequent vasculitis. FMF preceded PAN in all cases and was mostly controlled with low-dose colchicine treatment. Age at diagnosis of PAN (median, 36 years) was midway between patients with PAN and FMF described in a recent review (7), and those with idiopathic PAN (17). The prevalence of peri-renal hematoma in FMF-associated PAN could reach 50% (7, 18, 19) in the literature. Along this line, they affected 30% of our patients, and introduced the disease in all cases. Moreover, cutaneous manifestations were particularly frequent and heralded the disease in 60% of cases. Note, only one patient had livedo, unlike the classic cutaneous manifestations of PAN. Other findings were consistent with a literature review describing less weight loss, peripheral neuropathy, cardiac involvement, and more abdominal pain in patients with FMF-associated PAN (7). However, the proportions of joint and CNS signs were closer to that in idiopathic PAN (17). Overall, all these signs should raise the suspicion of PAN in a patient with FMF, and clinicians should be vigilant for the high risk of peri-renal bleeding. In this study, treatment of PAN was standard, except for three patients who received anakinra, a recombinant IL-1-receptor antagonist, resulting in resolution of clinical signs. Its efficacy suggests that IL1 may play a role in the pathophysiological mechanisms associated with vasculitis in these patients.

IgA vasculitis was the second most identified vasculitis in our cohort. FMF symptoms preceded vasculitis in 75% of patients; their FMF was mostly uncontrolled despite high colchicine levels, indicating active disease. These results may suggest a link between active FMF, an ongoing inflammatory state, and the triggering of IgA vasculitis (7). As such, clinicians should consider IgA vasculitis when they see purpura suggestive of small- or medium-vessel vasculitis in a patient with FMF. Our patients were older at the time of diagnosis of their vasculitis, had more renal and muscular involvement, and less fever, than has been described in IgA vasculitis associated or not with FMF (7). The prevalence of renal involvement in our series (83%) was close to that described by Audemard Verger et al. (70%) in their review of adult patients with IgA vasculitis (20). Abbara et al. reported an increased rate of intussusception (9%) in FMF-associated IgA vasculitis, which we did not observe in our cohort (7). However, 33% of patients presented digestive bleeding, which may be related to undiagnosed intussusception. Moreover, a low rate of IgA deposits was reported in FMF-associated IgA vasculitis (23%) (7). In this study, when histology was available, IgA deposits were present in 67% of cases, a rate like that described in patients with IgA vasculitis in the general population.

We described two patients with ANCA-associated vasculitis. In both patients, the diagnosis and management of FMF and vasculitis were challenging, given the overlap of FMF and vasculitis signs. Thus, although the association of FMF and ANCA-associated vasculitis could be fortuitous, we invite clinicians to evoke FMF in case of persistent compatible symptoms and/or unexplained chronic biological inflammatory syndrome, especially if the patient is of Mediterranean origin.

Few patients with FMF and unclassified vasculitis have been described so far (7). Almost half of them developed ischemic or bleeding complications. In this study, we described two patients with unclassified vasculitis; both developed such complications.

Behçet disease (BD) was proposed to be more prevalent in patients with FMF in a study by Schwartz et al. (6). BD shares clinical characteristics with FMF (21, 22). *MEFV* has been evaluated in several studies as a potential pathogenic gene of BD. There are contradictory results as some studies have shown an association of BD and FMF genes, whereas others did not (23–26). We did not identify any patient with both diseases, which could be due to the retrospective nature of the study, the absence of association between both diseases, or the low incidence of Behçet disease in France. In their literature review, Abbara et al. did not find a higher prevalence of BD in patients with FMF (7).

Besides vasculitis, patients with FMF could have an increased prevalence of various immune-mediated conditions, including spondyloarthritis (27, 28), psoriasis (29), hidradenitis suppurativa (30, 31), juvenile idiopathic arthritis (32, 33), multiple sclerosis (34), and inflammatory bowel disease (35, 36). The pathogenic mechanism of these associations remains unknown, particularly the role of *MEFV* mutations (37–42).

## Conclusion

In conclusion, this multicenter retrospective study confirms the predominant coexistence of IgA vasculitis and PAN with FMF in French multi-ethnic patients. The presence of a high frequency of bleeding in patients with FMF and PAN, IgA vasculitis, and unclassified vasculitis is intriguing. Whether FMF increases the frequency of bleeding, or whether those vasculitis are FMF-related and represent a severe form of FMF, especially in patients with two pathogenic mutations, is unknown.

## Data availability statement

The original contributions presented in this study are included in the article/supplementary material, further inquiries can be directed to the corresponding author.

## Ethics statement

This observational study was based on data extracted from the JIR-cohort, an international multicenter data repository established by the National Commission on Informatics and Liberty (CNIL, authorization number N°: 914677). Patients consented to be included in the JIR-cohort and were informed that data collected in medical records might be used for research study in accordance with privacy rules.

## Author contributions

SA wrote the first draft of the manuscript, collected the data, and performed the statistical analysis. SA, SG-L, GG, CB, OF, JB-M, and DS contributed to the conception and design of the study. SG-L and GG supervised the study. All authors contributed to the manuscript revision, read, and approved the submitted version.

## Footnotes

1
https://www.jircohorte.org/jircohort
